# 超高效液相色谱法测定大黄与铜离子络合能力

**DOI:** 10.3724/SP.J.1123.2022.06020

**Published:** 2023-04-08

**Authors:** Yapeng LIU, Wang ZHANG, Xinjie LIU, Huan WU, An ZHOU

**Affiliations:** 1.安徽中医药大学科研技术中心, 安徽 合肥 230038; 1. Research and Technology Center, Anhui University of Chinese Medicine, Hefei 230038, China; 2.中药复方安徽省重点实验室, 中药研究与开发安徽省重点实验室, 安徽 合肥 230012; 2. Anhui Province Key Laboratory of Chinese Medicinal Formula, Anhui Province Key Laboratory of Research and Development of Chinese Medicine, Hefei 230012, China

**Keywords:** 超高效液相色谱, 配位反应, 铜离子, 大黄, 肝豆状核变性, ultra-high performance liquid chromatography (UHPLC), coordination reaction, copper ion, rhubarb, hepatolenticular degeneration (HLD)

## Abstract

中药肝豆汤治疗铜代谢障碍疾病具有疗效显著、副作用小的优势,但由于缺乏评价铜离子(Cu^2+^)络合能力的技术手段,限制了肝豆汤配位活性成分的筛查和发现。为评价复方肝豆汤中主药大黄对铜离子的络合能力,该文通过优化大黄活性成分与铜离子配位反应条件,建立了超高效液相色谱法(UHPLC)测定中药大黄与铜离子络合能力的方法。样品经Agilent Eclipse Plus C18色谱柱(50 mm×2.1 mm, 1.8 μm)分离,以甲醇-0.1%(v/v)磷酸水溶液为流动相进行梯度洗脱,柱温30 ℃,流速为0.3 mL/min,检测波长为254 nm,进样体积为5 μL。研究通过UHPLC分析比较大黄与铜离子配位反应前后色谱峰变化,超高效液相色谱四极杆-飞行时间质谱(UPLC-Q-TOF-MS)鉴定大黄提取物中配位活性成分,计算大黄提取物与铜离子配位反应前后其色谱峰面积变化率来评价大黄中活性成分与Cu^2+^的络合能力。结果显示,大黄提取物与Cu^2+^在pH为9的体系中配位反应12 h达到平衡;鉴定出大黄提取物中20种主要成分,根据Cu^2+^配位率筛选出8种具有较强配位能力的化合物,其中没食子酸-3-*O*-*β*-D-(6'-*O*-没食子酰基)-吡喃葡萄糖苷、芦荟大黄素-8-*O*-*β*-D-葡萄糖苷、番泻苷B、l-*O*-没食子酰基-2-*O*-肉桂酰基-葡萄糖苷、大黄酚-8-*O*-*β*-D-(6″-*O*-乙酰)-葡萄糖苷、芦荟大黄素、大黄酸和大黄素的Cu^2+^配位率依次为62.50%、29.94%、70.58%、32.77%、34.61%、26.07%、28.73%和31.78%。该方法的建立可用于筛选与Cu^2+^具有络合能力的中药活性成分,同时可为其他中药与金属离子络合能力的评估和筛选提供借鉴。

## Abstract (en)

Gandou decoction (GDD) is a traditional Chinese medicine prescription that has been widely used to treat copper metabolism disorders in China with remarkable clinical effect and lower toxicity. However, evaluation of the complexation ability of copper ions is challenging, which hinders screening and discovery of coordinate active ingredients in GDD. An analytical method is needed to determinate the complexation ability of chemical constituents with copper ions. In this study, a rapid and accurate method based on ultra-high performance liquid chromatography (UHPLC) was developed to determine the complexing ability of rhubarb with copper ions. First, the optimal coordination reaction conditions between active ingredients of rhubarb and copper ions were determined. The samples were separated using an Agilent Eclipse Plus C18 column (50 mm×2.1 mm, 1.8 μm) with 5 μL injection volumes. The mobile phase was gradient eluted with methanol and water containing 0.1% (v/v) phosphoric acid at a flow rate of 0.3 mL/min. The detection wavelength was 254 nm and the column temperature was 30 ℃. Under the optimized chromatographic conditions, the rhubarb constituents were effectively separated. Next, peak areas of rhubarb were calculated before and after the coordination reaction between copper ions. The complexing ability of active ingredients in rhubarb with copper ions was evaluated by calculating the rate of changes of their chromatographic peak areas. Finally, ultra performance liquid chromatography-quadrupole time-of-flight mass spectrometry (UPLC-Q-TOF-MS) was used to identify the coordination active ingredients in rhubarb extract. Focusing on the coordination reaction conditions between active ingredients of rhubarb and copper ions revealed that the active ingredients of rhubarb and copper ions reached equilibrium by coordination reaction at pH 9 for 12 h. Methodological evaluation revealed the good stability and repeatability of the method. Under these conditions, 20 major components of rhubarb were identified by UPLC-Q-TOF-MS. According to the coordination rate of each component and copper ions, eight components with strong coordination were screened out (gallic acid 3-*O*-*β*-D-(6'-*O*-galloyl)-glucopyranoside, aloe emodin-8-*O*-*β*-D-glucoside, sennoside B, l-*O*-galloyl-2-*O*-cinnamoyl-glucoside, chysophanol-8-*O*-*β*-D-(6″-*O*-acetyl)-glucoside, aloe-emodin, rhein and emodin). The respective complexation rates of the components were 62.50%, 29.94%, 70.58%, 32.77%, 34.61%, 26.07%, 28.73% and 31.78%. Compared with other reported methods, the presently developed method can be used to screen the active ingredients of traditional Chinese medicines that have complexing ability with copper ions, especially in complex mixture systems. This study describes an effective detection technology for evaluating and screening the complexing ability of other traditional Chinese medicines with metal ions.

铜是人体所必需的微量元素之一,它在机体能量代谢、抗氧化等多种生理过程中发挥着重要作用,但铜过载可导致胆汁排铜功能紊乱,铜累积于肝、脑等部位易造成器官的损伤病变^[1,2]^。肝豆状核变性(HLD)是由常染色体ATP7B基因突变引发铜代谢障碍的一种隐性遗传病,其病因是Cu^2+^在体内累积不能排出^[3]^。以D-青霉胺为代表的铜离子络合剂为临床治疗HLD的一线药物,尽管铜离子络合剂在临床上具有较好疗效,但由于毒性、耐受性等缺点限制了其临床应用^[4]^。中药复方肝豆汤为治疗HLD的经验处方,课题组前期研究发现肝豆汤不仅具有显著的尿铜排泄效果,而且能促进肝功能恢复^[5⇓-7]^。大黄为肝豆汤的君药,大黄蒽醌类成分为该方入血、入肝主要活性成分^[8]^。含有酚羟基、羰基等基团的蒽醌类成分通常可与Cu^2+^形成配合物,促进Cu^2+^经尿液从体内排出从而发挥药效作用^[9]^。现有的研究由于缺少测定Cu^2+^络合能力的方法,限制了大黄中Cu^2+^配位活性成分的筛选和发现。因此,建立中药与Cu^2+^络合能力的快速、高效分析方法具有重要的意义。

目前,对于单体化合物与Cu^2+^络合能力的考察方法包括离子计法^[10]^、紫外可见分光光度法^[11]^、分子荧光法等^[12]^,上述方法虽可以有效检测化合物与金属离子络合能力,但无法对中药及复杂体系中与Cu^2+^络合能力的活性成分进行筛查和评价。为建立Cu^2+^络合能力在线检测方法筛选肝豆汤中主药大黄的Cu^2+^络合活性成分,本文对配位反应时间、浓度、pH等条件进行了优化,得到大黄提取物与Cu^2+^配位条件,采用超高效液相色谱(UHPLC)进行分离检测,建立了大黄与Cu^2+^络合活性成分的快速测定方法,并结合超高效液相色谱-四极杆-飞行时间质谱(UPLC-Q-TOF-MS)在相同的液相色谱条件下鉴定大黄提取物中配位活性成分,旨在为中药Cu^2+^配位活性成分筛选和评价提供有效的检测技术。

## 1 实验部分

### 1.1 仪器、试剂与材料

Agilent 1290超高效液相色谱仪(美国Agilent公司); Acquity UPLC I Class超高效液相色谱仪,Xevo G2-XS QTOF高分辨率飞行时间质谱仪,配有电喷雾离子源(ESI)(美国Waters公司); KQ-500DB型数控超声波清洗器(昆明市超声仪器有限公司); Sartorius BT 211D型电子天平(德国Sartorius公司); FW100型高速万能粉碎机(天津市泰斯特仪器有限公司)。甲醇为色谱纯(美国Fisher Chemical公司);甲酸为色谱纯(天津市光复精细化工研究所);屈臣氏超纯水;其余试剂均为分析纯。对照品芦荟大黄素(批号150730)、大黄酸(批号150904)、大黄酚(批号151028)均购于北京北纳创联生物技术研究所;大黄素(批号110756-201512)、大黄素甲醚(批号110758-201415)均购于中国食品药品检定研究院;大黄药材购于合肥市同仁堂大药房,经安徽中医药大学科研技术中心刘先华老师鉴定。

### 1.2 溶液的配制

#### 1.2.1 供试品溶液

将大黄药材粉碎,过40目筛,取大黄药材粉末0.5 g,精密称定,置于25 mL量瓶中,加甲醇溶解并稀释至刻度,称定重量。浸泡12 h,超声提取30 min,放置至室温,称重,补充甲醇至原重量,摇匀,滤过。精密吸取上清液2.50 mL,置于10 mL量瓶中,加甲醇定容至刻度,即得大黄供试品溶液,经0.22 μm微孔滤膜过滤,待测。

#### 1.2.2 混合对照品溶液

分别准确称取芦荟大黄素、大黄酸、大黄素、大黄酚和大黄素甲醚5种对照品,用甲醇溶解,配制成浓度为0.5 mol/L的标准储备液。分别移取5种标准储备液1.00 mL,置于10 mL容量瓶中,加甲醇定容至刻度,得到浓度为0.05 mol/L混合对照品溶液,备用。

#### 1.2.3 Cu^2+^标准溶液

准确称取适量CuCl_2_·2H_2_O,用甲醇配制成浓度为0.5 mol/L的Cu^2+^标准储备液,吸取适量上述Cu^2+^标准储备液,用甲醇配制成浓度为0.05 mol/L的Cu^2+^标准溶液,备用。

#### 1.2.4 混合样品溶液

各取1.00 mL混合对照品溶液于10 mL容量瓶中,依次加入0、0.25、0.50、1.00、2.00 mL不同体积的Cu^2+^标准溶液,分别用0.5%、1%(v/v)磷酸水溶液调节反应体系pH为4、5,另用0.5%、1%(v/v)氨水溶液调节反应体系pH为9、10,不加酸碱溶液反应体系pH为7,甲醇定容,摇匀,即得对照品混合样品溶液,经0.22 μm微孔滤膜过滤,待测。另取1.00 mL大黄供试品溶液,在pH为9的体系中加入过量Cu^2+^标准溶液,甲醇定容,摇匀,即得供试品混合样品溶液,配位反应12 h后,取上清液过0.22 μm微孔滤膜过滤,待测。

### 1.3 分析条件

#### 1.3.1 UHPLC

色谱柱为Agilent Eclipse Plus C18(50 mm×2.1 mm, 1.8 μm);流动相A:甲醇,流动相B: 0.1%(v/v)磷酸水溶液;流速:0.3 mL/min。梯度洗脱程序:0~5 min, 5%A~28%A; 5~15 min, 28%A~45%A; 15~30 min, 45%A~60%A; 30~40 min, 60%A~100%A。检测波长:254 nm;柱温:30 ℃;进样体积:5 μL。

#### 1.3.2 UPLC-Q-TOF-MS

色谱条件同1.3.1节。

质谱条件:离子源为电喷雾电离源,负离子模式;离子源温度110 ℃,扫描范围*m/z* 50~1200。毛细管电压和锥孔电压分别为2.5 kV和40 V;脱溶剂温度350 ℃;去溶剂化气流600 L/h;锥孔气流速50 L/h;流速0.30 mL/min。

## 2 结果与讨论

### 2.1 实验条件的优化

大黄蒽醌类化合物是肝豆汤中主要的入血、入肝成分,因此选取芦荟大黄素、大黄酸、大黄素、大黄酚和大黄素甲醚5种常见大黄蒽醌类成分优化大黄与Cu^2+^配位反应的实验条件。

#### 2.1.1 反应体系pH的优化

为优化配位反应的pH条件,考察不同pH对铜配合物生成的影响,以色谱峰面积变化的大小代表铜配合物生成量的多少判断适宜pH。分别在pH为4、5、7、9、10条件下,观察加入0、0.25、0.50、1.00、2.00 mL Cu^2+^标准溶液的对照品混合样品溶液的色谱峰面积变化,发现在pH为4、5条件下,色谱峰面积无明显变化,表明酸性体系不利于大黄与Cu^2+^的配位反应。而在pH为7、9、10条件下,对照品混合样品溶液色谱图的保留时间没有明显变化,峰面积随着Cu^2+^含量的增加而减小,与pH为7时相比,pH为9时各色谱峰面积变化较明显。通过比较色谱峰面积变化量,最适宜配位反应的pH顺序:碱性(pH 9>pH 10)>中性>酸性(pH 5>pH 4)。因此,选择pH为9作为最佳实验反应条件。

#### 2.1.2 反应时间的考察

配位反应是一个动态的化学平衡过程,确定最佳的反应时间可以准确地评估Cu^2+^配位率,分别在0、3、6、9、12、24 h观察同一对照品混合样品溶液的色谱峰面积变化,如图1所示,色谱峰面积随着时间增加逐渐减小,而12 h与24 h色谱峰面积变化无显著性差异,说明混合样品溶液在配位反应12 h达到平衡状态。因此,选择12 h为最佳实验反应条件。

**图1:**
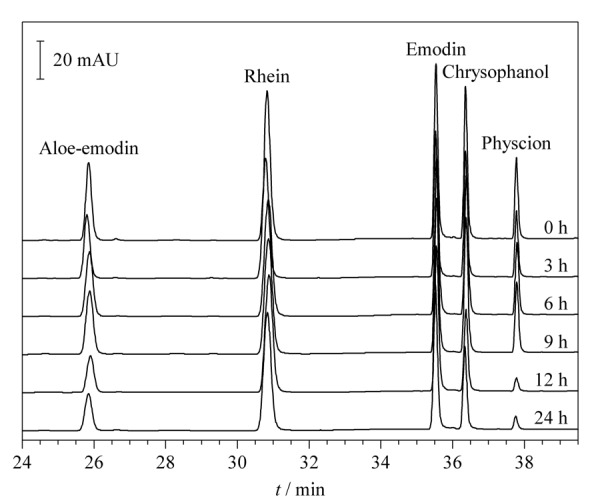
不同配位反应时间下对照品混合样品溶液的色谱图

#### 2.1.3 检测波长的选择

大黄蒽醌类化合物在250~280 nm波长范围内具有较好紫外吸收^[13]^。分别在254、260和280 nm处观察同一对照品混合样品溶液的色谱图,如图2所示,5种大黄蒽醌类成分在3处波长下整体均具有良好吸收,其中254 nm处各峰的紫外吸收较好。因此,选择254 nm作为实验条件的检测波长。

**图2:**
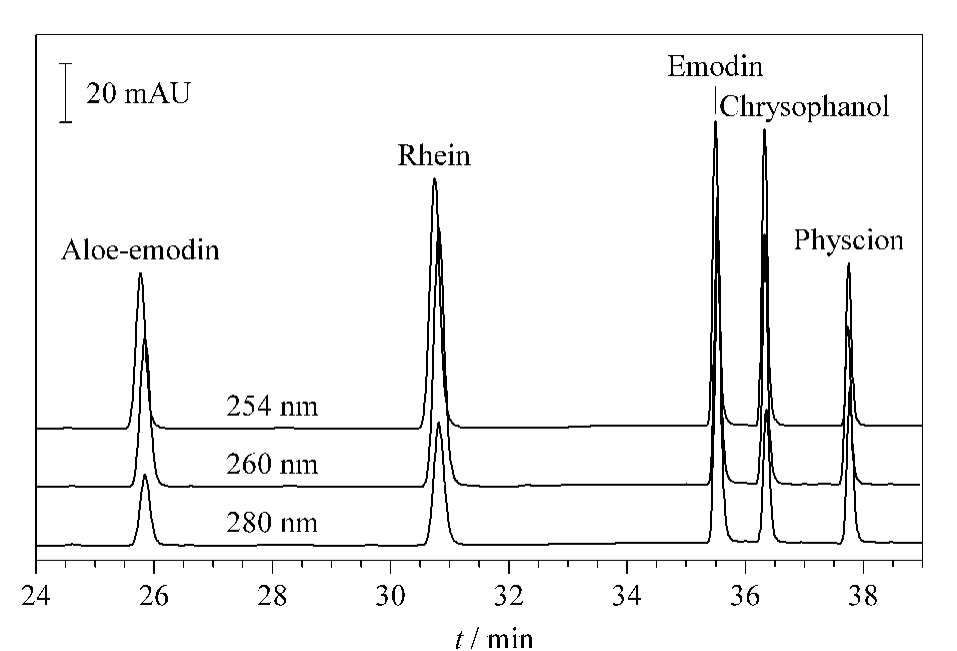
不同检测波长下对照品混合样品溶液的色谱图

### 2.2 大黄化学成分的鉴定

取1.2.1节制备的大黄供试品溶液,按1.3节色谱、质谱条件经UPLC-Q/TOF-MS测定,得到大黄提取物的总离子流色谱图,如图3所示。根据UPLC-Q/TOF-MS分析得到各个色谱峰的保留时间、精确质量数和碎片峰信息,对大黄中主要化学成分进行分析鉴定,确定了20种化学成分,详细结果见表1。

**图3:**
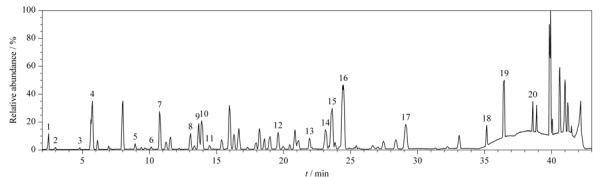
大黄提取物的总离子流色谱图

**表1:** 大黄化学成分的质谱数据及鉴定结果

No.	t_R_/ min	Observed (m/z)	Calculated (m/z)	Mass error/10^-6^	Ions (m/z)	Compound	Refs.
1	0.999	484.0913	484.0918	-1.03	483.0903, 439.1162, 331.0600, 313.0558	gallic acid 3-O-D-(6'-O-galloyl)-	[14,15]
						glucopyranoside	
2	2.208	332.0726	332.0723	0.90	331.0713, 271.0447, 169.0136, 125.0387	gallic acid-3-O-glucoside	[15,16]
3	2.500	332.0723	332.0723	0.00	331.0713, 271.0450, 169.0135, 125.0238	gallic acid-4-O-glucoside	[15,16]
4	5.303	290.0783	290.0790	-2.41	289.0743, 245.0813, 205.0500, 203.0709	catechin/epicatechin	[17,18]
5	8.305	442.0908	442.0900	1.81	441.0651, 331.0785, 289.2098, 169.9586	catechin-3-O-gallate	[17,18]
6	10.190	484.0765	484.0767	-0.41	483.0774, 331.0684, 313.0626, 169.0139	gallic acid-O-galloyl-glucoside	[14,15]
7	10.378	432.1056	432.1056	0.00	431.1048, 269.0504, 225.0550	aloe emodin-8-O-β-D-glucoside	[17,18]
8	12.433	446.0852	446.0849	0.67	445.0790, 283.0296, 239.0401, 211.0408	rhein-1-O-β-D-glucoside	[17,18]
9	13.352	848.2158	848.2164	-0.71	685.1567, 386.0983	sennoside C	[17,18]
10	13.871	862.1950	862.1956	-0.70	861.2141, 699.1530, 286.0977	sennoside B	[16,17]
11	14.504	462.1152	462.1152	0.00	461.1169, 313.0607	l-O-galloyl-2-O-cinnamoyl-glucoside	[15,18]
12	20.097	416.1116	416.1107	2.16	415.1116, 253.0539	chrysophanol-1-O-β-D-glucoside	[17,18]
13	21.495	432.1056	432.1056	0.00	431.1048, 269.0504	aloe-emodin-glucoside	[17,18]
14	24.341	458.1209	458.1213	-0.87	457.1229, 253.0539	chysophanol-8-O-β-D-(6″-O-acetyl)-	[17,18]
						glucoside	
15	25.065	446.1216	446.1213	0.67	445.1241, 283.0676, 240.0482	physcion-8-O-β-D-glucoside	[17,18]
16^*^	25.854	270.0536	270.0528	2.96	269.0504, 225.0545	aloe-emodin	[16,18]
17^*^	30.816	284.0332	284.0321	3.87	283.0296, 239.0352	rhein	[16,17]
18^*^	35.526	270.0533	270.0528	1.85	269.0504, 225.0597	emodin	[16,17]
19^*^	36.367	254.0579	254.0579	0.00	253.0515, 225.0545	chrysophanol	[16,17]
20^*^	37.775	284.0692	284.0685	2.46	283.0619, 240.0359	physcion	[16,17]

* Compounds were identified by the reference substance.

化合物1(*t*_R_=0.999 min)在负离子模式下产生的准分子离子峰[M-H]^-^的*m/z*是483.0903,在高碰撞能量下分别脱去一分子CO_2_、一分子没食子酸形成*m/z* 439.1162[M-H-CO_2_]^-^、*m/z* 313.0558[M-H-C_7_H_6_O_5_]^-^的碎片离子,对比相关文献资料^[14]^,推断化合物1为没食子酸3-*O*-*β*-D-(6'-*O*-没食子酰基)-吡喃葡萄糖苷。化合物2(*t*_R_=2.208 min)和化合物3(*t*_R_=2.500 min)在负离子模式下产生的准分子离子峰[M-H]^-^的*m/z*均是331.0713,其相对分子质量相比于没食子酸增加了162,推测两个化合物为没食子酸葡萄糖苷的两个同分异构体。化合物2的准分子离子在高碰撞能量下相继脱去一分子葡萄糖、一分子CO_2_形成*m/z* 169.0136[M-H-C_6_H_10_O_5_]^-^、*m/z* 125.0387[M-H-C_6_H_10_O_5_-CO_2_]^-^的碎片离子,其中*m/z* 125.0387[M-H-C_6_H_10_O_5_-CO_2_]^-^为没食子酸质谱鉴定的特征碎片离子,结合反相色谱系统中两者保留时间的差异,推测化合物2为没食子酸-3-*O*-葡萄糖苷,化合物3为没食子酸-4-*O*-葡萄糖苷^[15]^。化合物4(*t*_R_=5.303 min)在负离子模式下产生的准分子离子峰[M-H]^-^的*m/z*是289.0743,其产生的*m/z* 245.0813[M-H-CO_2_]^-^碎片离子是(表)儿茶素质谱鉴定的特征碎片离子,因此,推断化合物4可能为儿茶素或表儿茶素。结合质谱数据信息及文献参考^[16]^,化合物5、6、7、8、9、10、11推断为儿茶素-3-*O*-没食子酸酯、没食子酸-*O*-没食子酰基-葡萄糖苷、芦荟大黄素-8-*O*-*β*-D-葡萄糖苷、大黄酸-1-*O*-*β*-D-葡萄糖苷、番泻苷C、番泻苷B、l-*O*-没食子酰基-2-*O*-肉桂酰基-葡萄糖苷。大黄蒽醌类化合物在高能量碰撞中易断裂侧链取代基(如羟基、羧基、甲氧基等离子),通过对照品对比分析,化合物16、17、18、19、20推断为芦荟大黄素、大黄酸、大黄素、大黄酚、大黄素甲醚。化合物16(*t*_R_=25.854 min)和18(*t*_R_=35.526 min)在负离子模式下产生的准分子离子峰[M-H]^-^的*m/z*均是269.0504,化合物16在高能量碰撞下脱去一分子CO_2_形成*m/z* 225.0545[M-H-CO_2_]^-^的碎片离子,对比保留时间,推断化合物16为芦荟大黄素,化合物18为大黄素。化合物17(大黄酸)在负离子模式下产生的准分子离子峰[M-H]^-^的*m/z*是283.0296,在高能量碰撞下该化合物丢失侧链上羧基即脱去一分子CO_2_形成*m/z* 239.0352[M-H-CO_2_]^-^的碎片离子。结合化合物裂解规律及对比文献^[16]^,化合物12、13、14、15推断为大黄酚-1-*O*-*β*-D-葡萄糖苷、芦荟大黄素葡萄糖苷、大黄酚-8-*O*-*β*-D-(6″-*O*-乙酰基)-葡萄糖苷、大黄素甲醚-8-*O*-*β*-D-葡萄糖苷。

### 2.3 方法学考察

#### 2.3.1 仪器精密度

取1.2.4节制备的同一对照品混合样品溶液,配位反应12 h后,按1.3.1节色谱条件经UHPLC连续测定6次,计算各蒽醌类化合物峰面积RSD值,分别为0.20%~0.49%,说明仪器精密度良好。

#### 2.3.2 方法精密度

取1.2.4节平行制备的6份对照品混合样品溶液,配位反应12 h后,按1.3.1节色谱条件经UHPLC测定,计算各蒽醌类化合物峰面积RSD值,结果为0.43%~2.61%,说明方法精密度良好。

#### 2.3.3 稳定性

取1.2.4节制备的同一对照品混合样品溶液,配位反应12 h后,按1.3.1节色谱条件经UHPLC分别于0、2、4、8、12和24 h进行测定,计算各蒽醌类化合物峰面积的RSD值,结果为0.42%~0.55%,说明溶液稳定性良好。

### 2.4 大黄对铜离子的配位能力分析

取1.2.4节制备的供试品混合样品溶液与1.2.1节制备的大黄供试品溶液,按1.3.1节色谱条件经UHPLC进样分析,观察大黄供试品溶液与Cu^2+^配位反应前后的色谱峰面积变化。在大黄与Cu^2+^配位过程中,有少许褐色的大黄新配合物生成,且新生成的配合物不能在酸溶液(稀HCl)、碱溶液(NaOH)和有机溶液(甲醇等)中溶解;在氨水的碱性条件下,实验过程中未发现深蓝色的铜氨络合物,可能是生成的大黄新配合物稳定常数远大于铜氨络合物从而抑制了后者的生成。如图4所示,与未加入Cu^2+^的大黄供试品溶液相比,加入过量Cu^2+^的供试品混合样品溶液色谱图中部分峰的峰面积均具有不同程度的减少。各个化合物的峰对应的峰面积减少的值越大表明它与Cu^2+^反应生成的配合物的量越多,通过计算峰面积的减少量占总峰面积的百分比即是各个化合物与Cu^2+^的配位率。计算公式为:

*R*= $\frac{S_{0}-S}{S_{0}}$ ×100%

上述公式中,*R*代表Cu^2+^配位率,*S*_0_代表未加入Cu^2+^时供试品混合样品中各物质的峰面积,*S*代表加入过量Cu^2+^时供试品混合样品中各物质的峰面积。通过计算比较表1中各个化合物与Cu^2+^的配位率,筛选出8个与Cu^2+^具有较强配位能力的化合物,其中没食子酸-3-*O*-*β*-D-(6'-*O*-没食子酰基)-吡喃葡萄糖苷、芦荟大黄素-8-*O*-*β*-D-葡萄糖苷、番泻苷B、l-*O*-没食子酰基-2-*O*-肉桂酰基-葡萄糖苷、大黄酚-8-*O*-*β*-D-(6″-*O*-乙酰)-葡萄糖苷、芦荟大黄素、大黄酸和大黄素的Cu^2+^配位率依次为62.50%、29.94%、70.58%、32.77%、34.61%、26.07%、28.73%和31.78%。

**图4:**
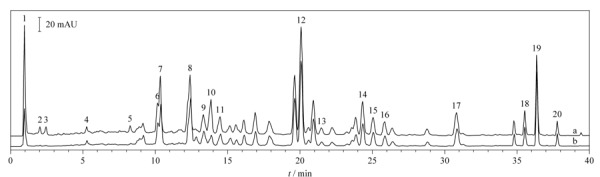
大黄提取物与Cu^2+^反应前后的色谱图

## 3 结论

本文通过优化大黄与Cu^2+^配位条件,建立了大黄与Cu^2+^配位能力的超高效液相色谱的测定方法,结合UPLC-Q-TOF-MS筛选鉴定出8种有较强Cu^2+^配位能力的大黄活性成分。该方法是对复杂样品中Cu^2+^络合活性成分筛选的有益尝试,可为HLD相关铜代谢障碍疾病药物筛选提供有效方法。
